# Adrenal insufficiency management in the pediatric emergency setting and risk factors for adrenal crisis development

**DOI:** 10.1186/s13052-023-01475-y

**Published:** 2023-06-06

**Authors:** Enrica Abrigo, Jessica Munarin, Claudia Bondone, Gerdi Tuli, Emanuele Castagno, Luisa de Sanctis, Patrizia Matarazzo

**Affiliations:** 1 Department of Pediatric Endocrinology, Regina Margherita Children’s Hospital, Piazza Polonia 94, Turin, 10126 Italy; 2 Postgraduate School of Pediatrics, University of Turin, Turin, Italy; 3 Department of Pediatric Emergency, Regina Margherita Children’s Hospital, Turin, Italy

**Keywords:** Adrenal insufficiency, Emergency, Adrenal crisis, Management, Pediatric age

## Abstract

**Background:**

In patients with adrenal insufficiency (AI), adrenal crisis (AC) represents a clinical emergency. Early recognition and prompt management of AC or AC-risk conditions in the Emergency Department (ED) can reduce critical episodes and AC-related outcomes. The aim of the study is to report the clinical and biochemical characteristics of AC presentation to improve their timely recognition and proper management in a ED setting.

**Methods:**

Single-centre, retrospective, observational study on pediatric patients followed at the Department of Pediatric Endocrinology of Regina Margherita Children’s Hospital of Turin for primary AI (PAI) and central AI (CAI).

**Results:**

Among the 89 children followed for AI (44 PAI, 45 CAI), 35 patients (21 PAI, 14 CAI) referred to the PED, for a total of 77 accesses (44 in patients with PAI and 33 with CAI). The main causes of admission to the PED were gastroenteritis (59.7%), fever, hyporexia or asthenia (45.5%), neurological signs and respiratory disorders (33.8%). The mean sodium value at PED admission was 137.2 ± 1.23 mmol/l and 133.3 ± 1.46 mmol/l in PAI and CAI, respectively (*p = 0.05*). Steroids administration in PED was faster in patients with CAI than in those with PAI (2.75 ± 0.61 and 3.09 ± 1.47 h from PED access, *p = 0.83*). Significant factors related to the development of AC were signs of dehydration at admission (*p = 0.027*) and lack of intake or increase of usual steroid therapy at home (*p = 0.059*). Endocrinological consulting was requested in 69.2% of patients with AC and 48.4% of subjects without AC (*p = 0.032*).

**Conclusion:**

children with AI may refer to the PED with an acute life-threatening condition that needs prompt recognition and management. These preliminary data indicate how critical the education of children and families with AI is to improve the management at home, and how fundamental the collaboration of the pediatric endocrinologist with all PED personnel is in raising awareness of early symptoms and signs of AC to anticipate the proper treatment and prevent or reduce the correlated serious events.

**Supplementary Information:**

The online version contains supplementary material available at 10.1186/s13052-023-01475-y.

## Background

Adrenal Insufficiency (AI) is related to a dysfunction of the adrenal gland (primary adrenal insufficiency, PAI), or a reduced secretion of pituitary adrenocorticotrophic hormone (ACTH) or CRH (central adrenal insufficiency, CAI) [1–3].

The most common causes of PAI in children are congenital adrenal hyperplasia (CAH, 70% of PAI), isolated autoimmune adrenalitis (Addison disease, 15% of cases) or are part of autoimmune polyglandular syndromes, sepsis, bilateral adrenal haemorrhage associated with meningococcemia, and various genetic syndromes, as X linked adrenoleukodystrophy, Allgrove syndrome, DAX 1-deficiency and others (Table 1) [1–4]. A PAI determines inadequate glucocorticoids and mineralocorticoids secretion, while androgen secretion may be normal, reduced or increased depending on the underlying disease [1, 5].

**Table 1:** Etiologies of Adrenal Insufficiency

Primary Forms	Secondary Forms
**Congenital**	**Congenital**
Congenital adrenal hyperplasia	Septo-optic dysplasia
Congenital adrenal hypoplasia	Maternal hypercortisolemia (hypothalamic suppression)
Familial glucocorticoid deficiency (ACTH unresponsiveness)	Corticotropin releasing hormone deficiency (hypothalamic disfunction)
Allgrove syndrome (alachrima, achalasia, ACTH unresponsiveness)	ACTH deficiency (pituitary dysfunction)
Metabolic disease	Pituitary aplasia/hypoplasia
Adrenoleukodystrophy	Prader-Willi syndrome
Smith-Lemli-Opitz syndrome	
Wolman disease	
Zellweger disease	
Mitochondrial disease	
**Acquired**	**Acquired**
Autoimmune adrenalitis (Addison disease)	Chronic steroid use
Isolated autoimmune adrenalitis	Abrupt steroid withdrawal
Autoimmune polyendocrine syndrome	Increased metabolic demand
Hemorrhage	Megesterol acetate (Megace) withdrawal
Birth trauma	Tumor
Trauma	Head trauma
Meningococcemia (Waterhouse-Friderichsen syndrome)	Burn injury
Medication	Radiation
Ketoconazole	Infiltrative disease
Etomidate	
Infection	
Cytomegalovirus	
Human immunodeficiency virus	
Fungal	
Tubercolosis	

The most common cause of CAI is iatrogenic suppression of the hypothalamic-pituitary adrenal (HPA) axis secondary to prolonged, high-dose glucocorticoid administration in chronic disease. Hypothalamic-pituitary diseases such as tumours, infiltrative lesions, congenital malformation, traumatic brain injury are the other frequent causes of CAI (Table 1) [6].

The clinical presentation of AI depends on the degree of impairment; the main symptoms include fatigue, nausea, muscle weakness and headache. Associated signs of AI can be dehydration, weight loss, lethargy, hyponatremia, hyperkalemia and hypoglycaemia. In case of PAI, elevated levels of ACTH and MSH for the absent negative feed-back from adrenals, lead to hyperpigmentation of the skin and mucous membranes, involving skin folds, armpits, groin, gums, and scars [1–3].

Diagnosis of AI is based on measurement of serum cortisol levels; a morning cortisol < 3 mcg/dl indicates AI, while cortisol levels > 15 mcg/dl rule out AI. For intermediate values it is mandatory to perform the dynamic test [6, 7].

The ACTH value can differentiate between PAI (ACTH above 2-fold upper normal values,) and CAI (inappropriately low-normal ACTH) [1, 5, 6].

PAI requires replacement therapy with both glucocorticoids (12–15 mg/mq/day) and mineralocorticoid (fludrocortisone 0.05–0.2 mg/day). In CAI, only cortisol replacement (10–12 mg/mq/day) is required, with no need for salt-retaining aldosterone replacement. The goal of therapy is to control the symptoms of AI with the lowest possible dose, without compromising the growth that is often observed in case of overtreatment [8, 9].

AC is a life-threatening emergency that requires prompt diagnosis and must be treated urgently. Literature data estimate an AC incidence of 5–10 episodes/100 patient-years and 1/200 episodes associated with a fatal outcome [10–13].

Stressful situations that can trigger AC include fever > 38 °C, intercurrent illness with vomiting, prolonged or massive diarrhea, infectious disease requiring antibiotics, acute trauma requiring medical intervention (such as fractures), anesthesia and associated surgical procedures [13, 14].

A serum cortisol concentration < 18 mcg/dL during acute illness can be indicative of AI. There is limited evidence to guide the optimal glucocorticoid dosing during stressful situations for children and adolescents with AI. Guidelines on stress cortisol requirements are based on the general evidence that conditions of maximal stress increase serum cortisol levels 2–3 times [11, 14].

After the diagnosis of AI, awareness and education about the symptoms and signs and treatment of cortisol deficiency to prevent AC should include the affected children and the family/caregivers and all healthcare professionals, from primary care physician to all ED personnel [14, 15].

Non-specific symptoms make the diagnosis of AC easily overlooked in the ED triage process; hypotension and hypoglycemia can develop suddenly, even after routine triage assessments, so providers must exercise a high index of suspicion for AC in any child who is at risk of AI [11, 12, 15].

Aim of this study is to provide useful information for emergency personnel to prevent, detect early and adequately treat AC in patients with AI during illness.

## Methods

Patients with AI followed by the Department of Pediatric Endocrinology and admitted to the PED of the Regina Margherita Children’s Hospital of Turin, Italy, between January 1st, 2013 and March 1st, 2022, were enrolled in this single-centre, retrospective, observational study.

The diagnosis of AI and ED visits were identified according to the criteria of glucocorticoid or ACTH deficiency reported in the ICD-10- CM system (PAI or CAI). Patients’ data were retrospectively retrieved from patients’ records and included demographic data (age, sex, ethnic origin, and residence), clinical information (age at diagnosis, specific diagnosis, home therapies) and ED admission features (number of visits and clinical characteristics of AC, triage priority code, electrolytes and glycaemic values, acute phase treatment, need for endocrinologist consulting).

According to ICD-10-CM system, AC was defined as acute clinical deterioration precipitated by glucocorticoid deficiency or rapid clinical improvement after glucocorticoids administration, with at least two of the following symptoms or signs, i.e. severe fatigue, nausea and vomiting, somnolence, hypotension/sinus tachycardia relative to age-related norms, hyponatremia, hyperkalemia or hypoglycaemia.

Statistical analysis was performed using GraphPad 7 Software (GraphPad Software, La Jolla, CA, USA) and T-test, Wilcoxon-Mann-Whitney test and Chi Square were applied to compare the clinical and demographic characteristics of the patients.

The study was conducted according to the guidelines of the Declaration of Helsinki and received the approval of the Hospital Ethics Committee. Informed parental consent was obtained from each participant.

## Results

**Fig. 1:**
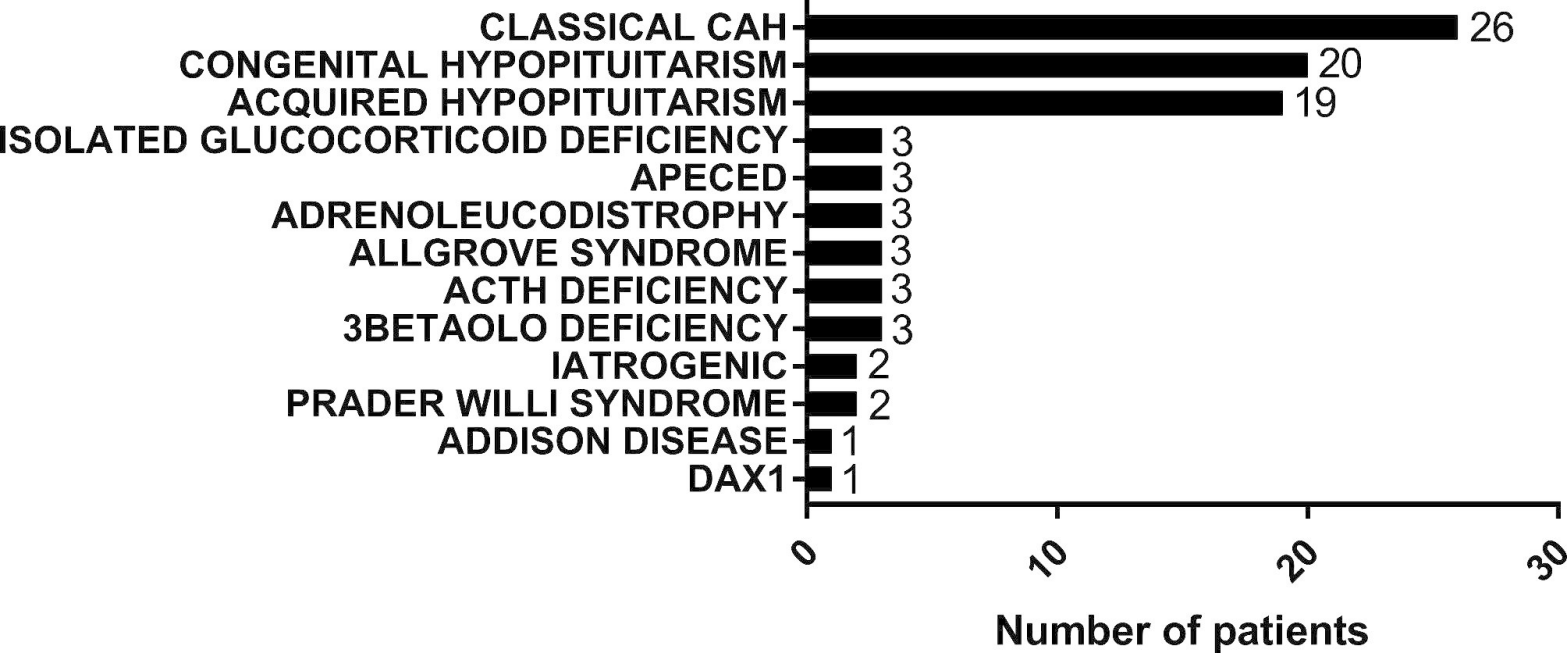
AI etiology in a cohort of pediatric patients referred in a tertiary department

The population with adrenal insufficiency (AI) followed by the Endocrinology Department of our Centre consisted of 89 children (56 boys, 33 girls). Of these, 44 were diagnosed as PAI (49.4%) and 45 as CAI (50.6%), due to different etiologies, represented in Fig. 1.

Demographic and clinical data of patients with PAI and CAI are represented in Table 2.

**Table 2:** Demographic and clinical features of patients affected by primary adrenal insufficiency (PAI) and central adrenal insufficiency (CAI).

	PAI	CAI	*p value*
**Gender (M/F)**	28/16	28/17	*0.89*
**Age at diagnosis (years)**	1.72 ± 0.58	5.32 ± 0.77	*0.0003*
**Age at ED access (years)**	5.56 ± 1.03	5.37 ± 1.04	*0.89*
**Steroid dose (mg/mq/day)**	13.7 ± 0.95	14.3 ± 0.73	*0.65*
**At home steroid management**			
**No change**	26	19	*0.94*
**Double oral dose**	6	5	
**Triple oral dose**	8	6	
**Intramuscular**	2	0	
**No therapy**	2	3	
**Symptoms duration - from onset to ED admission (hours)**	5 ± 1.4	28 ± 3.8	*0.005*
** N. of admissions**	44	33	
**Single admission**	7	4	*0.51*
**2 admissions**	8	4	
**3 admissions**	3	3	
**4 admissions**	3	3	
**Glucose value (mg/dl)**	73.6 ± 10.9	84.8 ± 12.4	*0.5*
**Sodium value (mmol/l)**	137.2 ± 1.23	133.3 ± 1.46	*0.05*
**Patients with adrenal crisis**	10	3	*0.06*
**Adrenal crisis episodes**	12	14	*0.57*

Mean age at diagnosis was 1.72 ± 0.58 and 5.32 ± 0.77 years for patients affected by PAI and CAI respectively.

During the study period, 35 patients accessed to the PED of the same hospital, 21 with PAI (47.7%) and 14 with CAI (31.1%) [*p = 0.1*]. AC was detected in 13/35 children (37,1%), 10/13 in children affected with PAI (77%) and 3/13 in children with CAI (23%). A total of 77 ED accesses were registered, 44 (57.1%) in patients with PAI and 33 (42.9%) with CAI. Among ED accesses 26/77 (33.8%) presented AC, with no difference between children with PAI and CAI forms.

Mean age at ED access was 5.56 ± 1.03 years for patients with PAI and 5.37 ± 1.04 years for patients with CAI (*p = 0.89*).

Regarding home therapy, the mean hydrocortisone dosage at ED admission was 13.7 ± 0.95 mg/m2/day in patients with PAI, while 14.3 ± 0.73 mg/m2 /day in patients with CAI (*p = 0.65*). Patients with PAI also required fludrocortisone treatment at a mean dosage of 100 *±* 50 mcg/day.

At diagnosis all patients were instructed to increase the hydrocortisone dosage in case of stressful situations. However, at the ED survey patients had not changed their usual steroid therapy in 58.4% of cases, 14.3% doubled and 18.2% tripled their usual dose, the 6.5% has missed steroid administration or was unable to take the usual steroid dose, with no difference between PAI and CAI patients. Intramuscular administration of hydrocortisone was required in 2 only patients.

At access to the ED, the yellow code was the main triage code assigned, due to moderately critical conditions of the patients (65.2%). In 93.5% of ED accesses, patients communicated to the triage setting to suffer from AI.

The main causes of hospitalization were signs of gastroenteritis, with vomiting and/or diarrhea (59.7%), followed by other causes including fever, hyporexia or asthenia (45.5%), neurological signs and respiratory disorders (33.8%), while only 2.6% and 1.3% had signs of dehydration or hypoglycaemia, respectively. Laboratory tests performed at ED access revealed a higher prevalence of hyponatremia and hypoglycemia than in patients without AC. The mean glucose value in patients with PAI was 73.6 ± 10.9 mg/dl, while 84.8 ± 12.4 mg/dl in patients with CAI (*p = 0.5*). The mean sodium value was 137.2 ± 1.23 mmol/l and 133.3 ± 1.46 mmol/l in PAI and CAI, respectively (p = 0.05).

During the ED access, 56% of patients received endovenous steroid therapy and 49.4% parenteral hydration; almost all patients with AC received parenteral hydration (12/13, 92.3%) with normal saline (2/12, 16.7%), dextrose solution (9/12, 75%) or both (1/12, 8.3%).

In the PED, steroids were administered earlier in patients with CAI than with PAI (2.75 ± 0.61 and 3.09 ± 1.47 h from PED access, *p = 0.83*).

Children who developed AC were treated with a slightly but not significantly lower dose of hydrocortisone than those who did not show AC (11.90 and 12.30 mcg/kg/day, respectively, p = 0.608).

The risk factors for the development of AC according to multivariate regression analysis were the signs of dehydration at presentation (*p = 0.027*) and failure to take or increase the usual steroid therapy at home (75% of patients with AC did not increase home therapy as recommended, *p = 0.059*).

No significant differences were observed in clinical features among patients who developed AC compared to patients who did not developed AC.

Patients with AC for whom pediatric endocrinological consulting was requested were significantly higher than those without AC (9/13, 69.2% and 10/22, 45.4%, respectively, p = 0.032). Almost all ED accesses of patients with AC resulted in hospitalization (24/26, 92.3%); the main diagnoses at ED discharge were dehydration and hypoglycaemia.

## Discussion

AI is a rare disease that requires replacement treatment to restore well-being and to prevent life-threatening events.

AC occurs with an acute deterioration in health status associated with acute hemodynamic imbalance, with hypotension or sinus tachycardia, or marked metabolic disorders, i.e. hyponatremia, hyperkalemia, or hypoglycemia. It represents the most severe complication of AI, which can occur in several stressful situations; its susceptibility varies among patients and unknown risk factors may potentiate the risk of occurrence [16].

The education of the pediatric patient and his family, as well as all the personnel working in the PED is essential to prevent and promptly treat this acute complication at home and upon arrival at the hospital during sick events. Previous findings suggest that physicians are the primary source for obtaining information about the disease and its complications. However, evidence show the need to improve their performances and awareness [16–18].

The incidence of AC in our cohort (3.2 episodes/100 patient-year) was in accordance with literature data [13, 14, 16]. Admission rates were higher among pre-school patients, as reported by previous studies, which may be explained by the frequent infections in this age group. Literature data describe a second peak of accesses to the ED in adolescents, attributable to the transition period from paediatric to adult services, to greater patient autonomy and the reduced adherence to therapy, exposing them to a greater risk of complications [17–19]. The same period of life is often characterized by the need for a therapy adjustment for the pubertal status.

To raise awareness of AC among healthcare professionals, consensus guidelines for patients with AI recommend that patients wear or carry medical alert devices, such as wallet cards, even though currently these alert devices are rarely used. In the present study, the patient informed the triage about the AI condition in 93.5% of accesses to the PED. Future use of a critical information note (CIN), as reported previously [15, 17], could provide details on the signs and symptoms to consider and recommendations to follow for the most appropriate management of AI patients in triage settings.

The patients described presented to the Hospital primarily for infectious diseases, mostly gastroenteritis, with no evidence of hemodynamic compromise. Of these, approximately half patients (44%) were later discharged after clinical evaluation, feeling well, without receiving additional steroid therapy during PED access, as most of them had doubled or tripled the habitual steroid dose at home prior to PED access. Literature data confirm that this could be the first approach to a patient with AI during stressful situations [11, 15].

In the cohort studied, a lower usual hydrocortisone replacement dose was observed in patients who developed AC; furthermore, home steroid therapy was not increased in 75% of cases. Based on epidemiological data, it has in fact been suggested that the increase in AC rates may be due to the current use of lower doses of glucocorticoids in patients with hypoadrenalism [5, 14, 18, 20].

Previous data report that children with PAI are at higher risk for AC than subjects affected by secondary forms [13, 14, 18–20], although in our cohort this difference was not significant, probably due to the low number of AC.

Eyal et al. suggested that younger age at diagnosis may be a risk factor for AC, with a higher incidence of AC in the first year of life [13]. In our cohort, age did not significantly influence the presentation of AC and there was no difference in the mean age at ED admission between primary and secondary forms.

The most common biochemical feature of AC was hyponatremia, occurring in 53.8% of the subjects, with lower mean sodium value in primary than secondary forms (p = 0.05), followed by hypoglycemia (38.5%), in accordance to previous studies on children and adults, especially on patients with PAI [10, 11, 18–20].

Treatment of AC is effective when given promptly. In the PED setting, steroid administration was faster in CAI than PAI patients, probably for more severe and acute presentation and the recognized underlying disease in the latter form. Findings from a previously published survey result from AI adult patients in the United Kingdom reported a time limit of 30 min from ED arrival to hydrocortison administration. Additional interventions, such as triage protocols for patients with AI, might be useful to expedite hydrocortison administration after clinical evaluation [15, 18].

The risk factors for the development of AC were dehydration at presentation and the failure to take or increase habitual steroid therapy at home.

Appropriate management requires prompt recognition of the clinical signs, symptoms and biochemical profile of AI and AC triggers [21].

To our knowledge, this is the first study that reports the clinical features of AI patients accessing a PED and evaluates risk factors for AC. The strengths of the study are that the information is based on the patients’ medical records and not on their self-reports and that the cohort includes patients with both primary and secondary AI.

Limitations are the retrospective nature of the study, with potential under- or over-reporting of AC. Certainly, further prospective and multicenter studies are needed to analyze the accesses of patients with AI in the PED, to establish guidelines for the management of these patients with potentially life-threatening events in emergency settings.

## Conclusions

Children with AI may present to the PED setting with an acute life-threatening condition that requires prompt and effective recognition and management to avoid serious complications.

Our study confirms the previously observed high complexity of this disease, especially in the emergency setting, and added more information on the clinical characteristics of these patients and some red flags to consider.

While patient and family education is the key to successful prevention and treatment of AC crises at home in AI patients, it should be emphasized that raising information and awareness of this rare but potentially fatal complication in all healthcare professionals working in emergency settings is equally critical, to recognize it early and treat it adequately.

### Electronic supplementary material

Below is the link to the electronic supplementary material.

Supplementary Material 1

## Data Availability

All data are available upon request to the corresponding author.
