# Supplementary material for: Adrenal insufficiency management in the pediatric emergency setting and risk factors for adrenal crisis development
Source: Ital J Pediatr. 2023 Jun 6;49:63. doi: 10.1186/s13052-023-01475-y (PMC10242818; doi:10.1186/s13052-023-01475-y)
Supplement: Supplementary file 1 — Supplementary Material 1 [file 13052_2023_1475_MOESM1_ESM.docx]

Reviewer #1: Abrigo et al reported in this paper the last 9 years experience of a pediatric Centre in the management of adrenal insufficiency  in emergency department and evaluated the risk factors for adrenal crisis development. They briefily discussed the causes of AI and reported and discussed the results of their retrospective study.

The paper well reported a considerably high number of cases, managed in a single center and  raises important alerts for pediatric endocrinologists, general pediatricians and pediatricians of the Emergency Departments about the possibility of an adrenal crisis in patients with AI.

I think the paper should be further improved by addressing some important issues. According to the methods, some other data should be available as: type of adrenal insufficiency, age at the onset and age at presentation to the emergency department, number of the admissions to the ED by single patient, duration of symptoms prior to the admission to ED, type of management ( double or triple the dose, intramuscular hydrocortison etc)  , all these features should be considered and tested as risk factors for the development of the adrenal crisis by a performing a multiple regression analysis. I am not completely convinced that the colour of the triage code (yellow or red) could be literally considered as a risk factor. On the other hand the age of the patients could represent an important risk factor.

The authors could evaluate also if there are statistical differences for the above mentioned features among the patients with or without adrenal crisis.

In many cases the authors reported the results with p values that are not statistically significant, I suggest to separate or explain in the text the statistical significant results from the not significant ones. A table or two would help to do this.

**Response to reviewer 1:**

As suggested we added in the Result section the data about type of adrenal insufficiency, age at the onset and age at presentation to the emergency department, number of the admissions to the ED by single patient, duration of symptoms prior to the admission to ED, type of management .

All data are represented in table 2.

Multiple regression analysis was performed including the above criteria and significant difference was observed only for signs of dehydration at presentation and failure to take or increase the usual steroid therapy at home.
